# A second generation SNP and SSR integrated linkage map and QTL mapping for the Chinese mitten crab *Eriocheir sinensis*

**DOI:** 10.1038/srep39826

**Published:** 2017-01-03

**Authors:** Gao-Feng Qiu, Liang-Wei Xiong, Zhi-Ke Han, Zhi-Qiang Liu, Jian-Bin Feng, Xu-Gan Wu, Yin-Long Yan, Hong Shen, Long Huang, Li Chen

**Affiliations:** 1 Key Laboratory of Exploration and Utilization of Aquatic Genetic Resources Certified by Ministry of Education, College of Fisheries and Life Science, Shanghai Ocean University, 999 Hucheng Huan Road, Pudong New Area, Shanghai, 201306, China; 2 Shanghai Fisheries Technical Extension Station, Shanghai Fisheries Research Institute, Shanghai 200433, China; 3 Shanghai Mudbeach Institute of Biological Resource Exploitation, Shanghai, 202150, China; 4 Biomarker Technologies Corporation, Beijing 101300, China

## Abstract

The Chinese mitten crab *Eriocheir sinensis* is the most economically important cultivated crab species in China, and its genome has a high number of chromosomes (2n = 146). To obtain sufficient markers for construction of a dense genetic map for this species, we employed the recently developed specific-locus amplified fragment sequencing (SLAF-seq) method for large-scale SNPs screening and genotyping in a F1 full-sib family of 149 individuals. SLAF-seq generated 127,677 polymorphic SNP markers, of which 20,803 valid markers were assigned into five segregation types and were used together with previous SSR markers for linkage map construction. The final integrated genetic map included 17,680 SNP and 629 SSR markers on the 73 linkage groups (LG), and spanned 14,894.9 cM with an average marker interval of 0.81 cM. QTL mapping localized three significant growth-related QTL to a 1.2 cM region in LG53 as well as 146 sex-linked markers in LG48. Genome-wide QTL-association analysis further identified four growth-related QTL genes named LNX2, PAK2, FMRFamide and octopamine receptors. These genes are involved in a variety of different signaling pathways including cell proliferation and growth. The map and SNP markers described here will be a valuable resource for the *E. sinensis* genome project and selective breeding programs.

As a powerful genomic tool, genetic linkage maps have been widely utilized for mapping of quantitative trait loci (QTL), positional cloning of candidate genes, anchoring whole-genome scaffolds sequence, and comparative genomics and evolution studies^1^,^2^,^3^. A genetic linkage map is constructed with polymorphic DNA markers, which are genotyped in a family and grouped in the linear order along chromosomes based on the meiotic recombination frequency. The type and number of DNA markers is an important determinant of the resolution and density of a genetic linkage map. The innovation of DNA marker technology always brings forth dramatic enhancement of linkage map resolution. Especially the advent of high-throughput next-generation sequencing (NGS) technology has revolutionized the way of DNA markers discovery. Since the discovery of DNA marker in the 1980s, various types of DNA markers have been developed in many ways. Generally, molecular markers can be classified into three major types: (1) hybridization-based markers such as restriction fragment length polymorphisms (RFLPs)^4^; (2) PCR-based markers like random amplification of polymorphic DNA (RAPD)^5^, amplified fragment length polymorphism (AFLP)^6^, simple sequence repeats (SSRs); (3) sequence-based markers: Single nucleotide polymorphism (SNP)^7^, which is a DNA sequence variation caused by only one nucleotide. Although SNPs are being less polymorphic than SSR markers due to their biallelic nature, SNPs are the most abundant and uniformly distributed in the genomes. Compared to low-throughput markers based on size discrimination or hybridization, SNP is amenable to high-throughput technology, such as NGS technologies, which makes it possible to rapidly identify a large number of SNPs in the genome. However, the whole genome resequencing using NGS technologies for SNP discovery and genotyping is only suitable for a few model species that have a simple genome or have reference genomic sequence. It is not applicable for the majority of species with complex genomes, e.g. highly repetitive genomes, and no prior genomic information. To overcome these obstacles, several genome complexity reduction techniques have been developed over the years, including complexity reduction of polymorphic sequences (CRoPS)^8^, restriction site-associated DNA sequencing (RAD-seq)^9^, genotyping-by-sequencing (GBS)^10^, sequence-based genotyping (SBG)^11^. Through complexity reduction, a large portion of repetitive sequences was filtered out and, thus, these methods can be applied in SNPs discovery in a genome-wide fashion and genotyping of large genomes without the need of a reference genome. Recently, a modification of RAD sequencing method, termed specific-locus amplified fragment sequencing (SLAF-seq), has been reported by Sun *et al*.^12^. Unlike the RAD-seq method, the SLAF-seq involves size selection of restriction site-associated fragment for excluding random interruption of the DNA, and the selected SLAFs fragment is measured by pair-end sequencing on double barcode genotyping systems. Therefore, this approach is more efficient and cost-effective in SNP screening and genotyping than RAD-seq^12^, and has been increasingly used to develop high-density genetic maps in a variety of plants and animals^1^,^2^,^13^,^14^, especially in species without reference genome information.

In aquaculture species, many high density linkage maps for fishes^15^,^16^ and shellfishes^3^,^17^ have been developed using SNP and/or SSR markers, and the quantitative trait loci (QTL) for important traits including sex^18^,^19^,^20^ and growth^17^,^18^,^21^ traits have been identified. A large number of gene-associated SNPs derived from ESTs and RNA-seq dataset have also been discovered^22^,^23^,^24^,^25^,^26^ which could be great benefit for the breeding program and whole genome association studies. As for shrimps and crabs such as *Penaeus monodon*^27^, *Fenneropenaeus chinensis*^28^, *Litopenaeus vannamei*^29^ and *Portunus trituberculatus*^30^, previous linkage maps were constructed mainly relying on dominant markers AFLP or RAPD. Only several SNP and/or SSR-based high dense linkage maps have been recently reported^31^,^32^,^33^,^34^. The Chinese mitten crab is the most economically important cultivated crab species in China. There are three major natural populations that distribute in the basin of the Liaohe, Yangtze and Oujiang rivers in China. Southern population from Yangtze river basin was thought to have better growth performance and has become widespread cultivation. Through years of traditional phenotype-based selective breeding, several improved varieties of *E. sinensis* have been obtained based on selection of northern and southern populations^35^,^36^. The selected fast-growing populations of the crab promoted the crab farming industrial development to some extent. However, the current culture industry of the mitten crab still faces many problems, such as sexual precocity causing a great loss to farmers^37^. Precocious crabs reach maturity one year earlier at a small size. On the other hand, little is known about the genetic basis for most of the traits related to commercial production. Most of previous genetic studies on the mitten crab were performed focusing on population genetics^38^,^39^. In recent year, transcriptomic analysis using RNA-Seq technology was conducted on the immunity, molt, metamorphosis and reproduction^38^,^40^,^41^,^42^. A high-density linkage map of the mitten crab including 10,358 markers was developed on a northern population with 2b-RAD method^32^, but the markers identity is obscure because their sequences of the nucleotide have neither been described nor deposited in the data bank. The crab whole genome sequencing work has just been finished in our lab. We previously constructed a first generation SSR-based linkage map of the mitten crab using an F1 full-sib family from an intercross between Liaohe (northern) and Yangtze (southern) river populations in China^31^. However, since the generated sex-specific maps consisted of only 457 and 466 SSR markers, and included many triplet and doublet, which made it impossible to develop an integrated sex-average map. Obviously, generation of an integrated genetic map including more markers will provide a valuable framework for genome sequence assembly towards elucidation of the crab genome, and accelerate the breeding programs. Here, we used the same full-sib family as the mapping population and randomly selected 147 F1 offsprings for SNP discovery and genotyping based on SLAF-seq. Finally, we constructed a high-density SNP and SSR integrated genetic map of the mitten crab including 18,309 molecular markers. A growth-related genomic region was localized by QTL mapping and genome-wide QTL-association analysis.

## Results and Discussion

### Mapping family

The selection of mapping parents for establishing a mapping family is a key step to construct a high-density map. Backcross, F2, and recombinant inbred lines are the most commonly used populations for linkage mapping. For most shrimps and crabs, however, their life span is only about one or two years, which makes it unfeasible to develop a backcross population. Pseudo F1 populations are usually used as a mapping family^2^,^28^,^30^,^43^,^44^. In the pseudo-testcross procedure, two highly heterozygous parents with significant genetic difference are selected for hybridizing to produce a set of F1 progeny. Previous studies showed that the mitten crabs from northern and southern populations display trait divergence, such as body size^45^, and experiments from cultured crabs have shown that this differentiation is genetically based^46^,^47^. Yangtze crab and Liaohe crab had different gene pools and were embodied by different allelic frequencies in their genomes^39^. With this in mind, we established a mapping family from an intercross between Yangtze crab and Liaohe crab. Thousands of progeny were produced from a pair of parents, making it possible to obtain accurate estimates of marker positions in the map.

### Discovery of SLAF markers and genotyping

**Figure 1:**
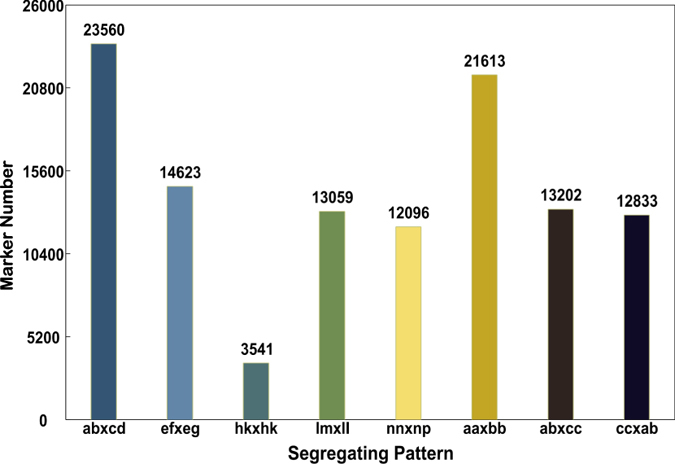
Number of SLAF markers for eight segregation patterns.

The mitten crab *E. sinensis* genome has a large number of chromosome (2n = 146)^48^. Sufficient marker is an essential prerequisite for construction of a dense genetic linkage map. To this end, we established SLAF libraries for high-throughput sequencing, which generated a total of 123,599,603 pair-end reads (Table 1). Among them, the high-quality bases (Q score > 30) ratio was 85.8% and guanine-cytosine (GC) content was 44.3%. After sequence alignment and clustering (for details see Materials and Methods), and discarding the low-depth and repeat-suspicious SLAFs, a total of 235,619 high-quality SLAFs were defined, of which 194,887 were detected in the female parent, and 200,452 were detected in the male parent. The reads numbers for SLAFs were 5,771,500 and 6,360,679 with an average coverage of 29.6-fold and 31.7-fold for each SLAF in the female and male parents (Table 1), respectively. In the 147 progeny of the mapping population, the average number of SLAFs was 107,758 with a coverage of 3.15-fold in each progeny. Among the 235,619 high-quality SLAFs that were defined, 127,677 were polymorphic, 105,981 were non-polymorphism and other 1961 were repetitive SLAFs. The polymorphism rate of these high-quality SLAFs was 54.2% similar with SSRs polymorphic rate (51.9%) detected previously in the same population^31^, implicating some genetic differentiation between germplasm resources of southern and northern populations. After removing the SLAFs with no parent information, 114,527 of these polymorphic SLAFs were retrieved and classified into eight segregation patterns (Fig. 1). As shown in Fig. 1, over 21,613 of markers were homozygous in two parents with genotype aa or bb, which belong to unsegregated patterns in the progeny. After filtered out these unsegregated markers and low quality SLAF markers with average sequence depths less than 10-fold in parents and integrities less than 90% in progeny, 20,803 markers conformed to the F1 population segregation codes, including ab × cd, ef × eg, hk × hk, lm × ll, and nn × np (Table 2). At a MLOD threshold of 5.0, 17,680 of these 20,803 markers were defined as effective markers and used for subsequent genetic linkage mapping. Average sequencing depths of these 17,680 markers were 59.34-fold, 49.63-fold and 4.26-fold in the female parent, the male parent and each progeny, respectively (Table 3). All the effective SLAF marker sequences were presented in Supplementary Table S1. In comparison with 10,358 markers previously identified within a northern population^32^, the resultant effective primer numbers in our present study increased largely when genotyped in the intercross family between northern and southern populations. The abundance of polymorphic markers implied the high heterozygosity and complexity of the crab genome.

### Construction of genetic linkage maps

**Figure 2:**
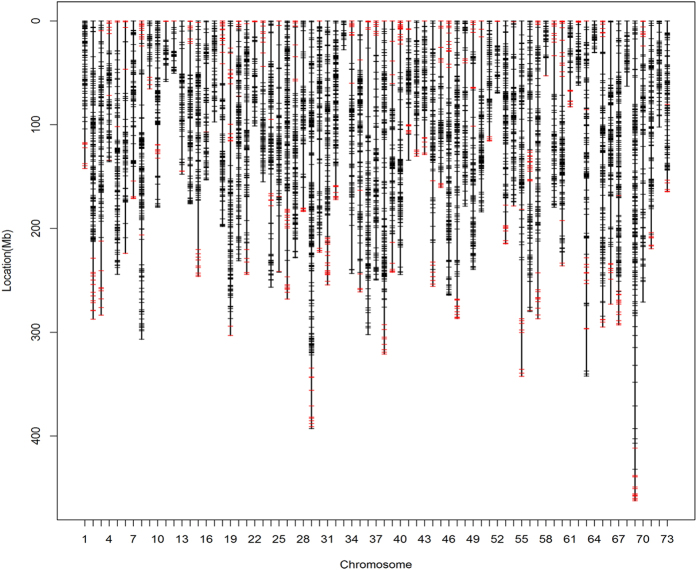
Distributions of SLAF (black bar) and SSR (red bar) markers in the linkage groups.

To develop a SNP- and SSR-based integrated map, the newly developed effective SLAF markers were combined with 629 SSR markers from the first-generation linkage map for linkage analysis^31^. By means of the two way pseudo-testcross strategy, sex-specific linkage maps were first constructed for each parent at a LOD threshold of 5.0, resulting in 73 linkage groups consistent with the haploid chromosome number of *E. sinensis*. All of these markers were mapped onto the genetic maps. The female map contained 12,332 markers (520 SSR and 11,812 SLAF) and spanned 15467.37 cM with an average interval of 1.25 cM, while the male map contained 12,699 markers (508 SSR and 12,191 SLAF) and spanned 13817.44 cM with an average interval of 1.09 cM (Table 4). The sex-specific linkage maps were further integrated into a sex-averaged map (Fig. 2; Supplementary Figure S1). The sex-averaged map consisted of 18,309 markers (629 SSR and 17,680 SLAF) and spanned 14,821.92 cM with an average interval of 0.81 cM (Table 4). By using two calculation methods for genome size estimation^49^,^50^, the genome map length was estimated to be 14069.79 cM for male, 15733.29 cM for female, and 15025.28 cM for the sex-averaged. Genome coverage was determined based on the ratios of the observed and estimated map lengths. Thus, the genome coverage of the male, female, and sex-average linkage map was 98.30%, 98.30% and 98.64%, respectively. Considering that the present map lengths are much longer than the map created by Cui *et al*.^32^ using only SNP markers^32^, we tried to use only SLAF markers for reconstruction of the map (Supplementary Figure S2). The total length of the reconstructed map shorten approximate 5000 cM, indicating that the extra length resulted from the integration of SSR markers rather than genotyping error. The increased length is most likely due to the uneven distribution of SSR markers in LGs. As shown in Fig. 2, most of SSR markers located in the terminus regions of LGs such as LG1-6, which significantly extended the LGs lengths. In comparison with 10,358 markers in the previous map^32^, a total of 18,308 markers were grouped in the present map. It is obvious that the extra length of the map is also due to huge increase in the number of mapped markers. Additionally, unlike 2b-RAD method, the SLAF-seq approach sequenced only regions near the enzyme sites. The uneven distribution of enzyme sites caused the uneven distribution of SNP markers along the linkage map, which could also result in longer map. To avoid artificial inflation of map lengths, we set strict criteria for the screening and genotyping of SNP markers as mentioned above. The high coverage and integrity for each resultant SNP locus greatly reduced genotyping errors and ensured the accuracy of linkage analysis.

### Segregation distortion markers on the map

Segregation distortion is a ubiquitous phenomenon that is defined as a deviation of the observed genotypic frequency from representative Mendelian segregation ratios. This deviation was thought to be caused by biological factors such as gametic and zygotic selection, and/or environmental factor^51^,^52^,^53^. Biological segregation distortion is always associated with a cluster of skewed markers within a chromosomal region, termed as segregation distortion region (SDR)^51^,^52^. In the present study, of 18,309 mapped markers, 2,879 markers exhibited significant segregation distortion from Mendelian expectations (P < 0.05) on the sex-averaged map (Supplementary Table S2), and were widely distributed on every linkage group. A total of 209 SDRs were found on 66 LGs. The average frequency of segregation distortion markers (15.72%) was similar to that being observed previously in the same mapping population when genotyped using SSR markers only^31^, implicating that the segregation distortion could not be caused by experimental artifacts. The distribution of distorted markers varied greatly between and within LGs on the map. The number of segregation distortion markers in linkage group ranges from 4 to 197. The frequency of segregation distortion markers on LG14, LG21 and LG23 was much higher than other LGs at 63.74%, 65.89% and 62.07%, respectively. The degree of linkage between adjacent markers of each LGs was presented as ‘Gap < 5 cM’ in Supplementary Table S2, which ranged from 90.55% to 100% with an average of 96.13%. Although the molecular mechanism of segregation distortion remain uncovered, accumulating data showed that segregation distortion markers did not have a large effect on QTL mapping, instead, distorted markers for linkage map construction could increase the genome coverage of the genetic map, and help to improve the detection of linked QTLs^53^,^54^.

### Differences in recombination rates between sexes

Different recombination rates between sexes have been reported in many species in which meiosis suppression occurs in one sex^55^,^56^,^57^. Generally, the heterogametic sex (XY or ZW) typically has a less recombination rate. As shown in Table 4, the female and male ratio for the recombination rates of the sex-specific maps was 1.12:1 in general. Further, the sex recombination ratio for shared markers was also calculated as previous studies^58^. Across all of the LGs, a total of 6726 informative markers was shared between the female and male maps (Supplementary Table S3). Based on the shared markers, the total length of the linkage map was 15367.58 and 13641.96 cM in the female and males, respectively. Thus, the female and male ratio of the recombination rate of shared markers was 1.13:1 (Supplementary Table S3), indicating that the sex recombination ratio of shared markers was similar with those of all the markers in the sex-specific maps (Table 4). This result is consistent with those of the first SSR-based linkage map in the mitten crab^31^ and in other decapod species^30^. Though the total lengths of female and male maps are similar, significantly different recombination ratios between sex-specific maps were observed in some LGs. The female and male ratios for the recombination rates of shared markers for each linkage group ranged from 0.50:1 in LG12 to 2.41:1 in LG41. The highest recombination rate ratios (greater than 2.0) were observed in LG41 2.41:1, 2.04:1 in LG48, while the lowest (less than 0.6) were LG12 0.50:1, and 0.54:1 in LG34 (Supplementary Table S3).

### QTL mapping for phenotypic sex

**Figure 3:**
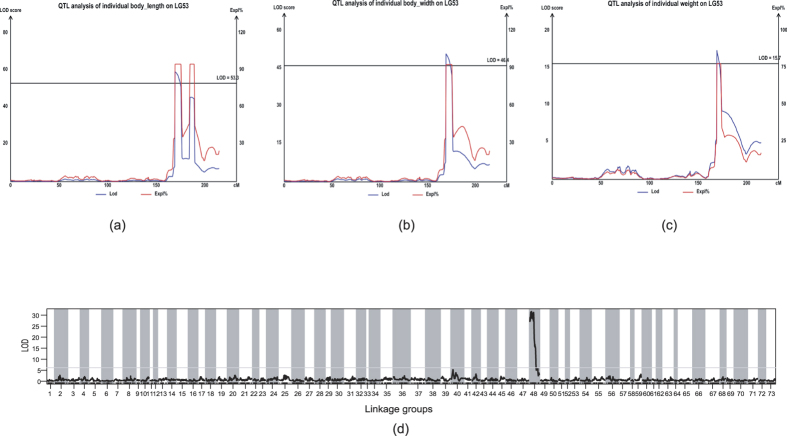
QTL mapping for the growth traits including body length (**a**), width (**b**) and weight (**c**), and sex (**d**) in *E. sinensis*. The blue and red curves indicate logarithm of odds (LOD) scores and percentage of explained phenotypic variance (Expl) of SLAF markers against their genetic position on LG53.

The genetic mechanism of sex determination in crabs is controversial. Early karyotypic analysis revealed a XY-XX sex determination system in several crabs^59^,^60^. However, recent linkage analysis in a family indicated that sex-linked markers were heterozygous and segregated only in the female parent, suggesting a ZW-ZZ system in the mitten crab^31^. Unfortunately, these sex-linked markers have not been converted into sex-specific markers to support the putative system. In this study, 146 markers linked with phenotypic sex were assigned to LG48 (Fig. 3d), on which significant differences in recombination rates between the two sexes was observed as mentioned above. A less recombination ratio was found in the male crabs than the female crabs, implicating the male might be the heterogametic sex (XY) since the heterogametic sex usually has a less recombination rate. Surprisingly, of the 146 sex-linked markers, no skewed markers were detected towards either female or male parent judged on the sex ratio of the segregation markers (Supplementary Table S4). Thus, our data cannot support a XY-XX or a ZW-ZZ system. Further, we performed genome-wide analysis using these sex-linked markers, no known sex-related gene was found in the scaffold anchored with LG48. The potential sex determination genes including *sxl, tra* and *dmrt* were detected in the scaffolds anchored to other LGs (data not shown). Similar result was also reported previously in the searching of the crab transcriptoms^32^. In addition, we ever made a parallel screen the mitten crab and prawn genomes for sex-linked markers using the method of amplified fragment length polymorphism (AFLP). Two reliable female-specific AFLP fragments were successfully detected in the prawn^61^, while no sex-specific marker was identified in the mitten crab even if much more AFLP-primer combinations were used in the screening^62^. Accordingly, the sex determination mechanism in the crab could be more complicated than the prawn. To draw a robust conclusion on the sex determination system of the crab, more families and populations should be employed in sex-linkage analysis and the sex-specific marker needs to be isolated for validating the sex chromosomes.

### QTL mapping and association analyses for major growth traits

QTL mapping analysis was performed for the growth traits including body length, width and weight (Supplementary Table S5). Interestingly, all the growth-related QTL at genome-wide level were detected in the same linkage group LG53 (Fig. 3, Table 5), indicating that LG53 may represent a major chromosome controlling the growth of *E. sinensis*. Three closest markers, Marker253119, Marker01950 and Marker209500, were localized to a 169.43–170.60 cM region significantly associated with each of the growth traits (length, width and weight), explaining 78.5–95.5% of the phenotypic variance (Table 5). The high phenotypic variance explained by these loci implicates that these loci are major QTL for the crab growth performance. Generally, the phenotypic variance explained (Expl) by QTL is strongly affected by mapping population size and individual gene effects. If two QTLs are linked in the repulsion phase on one chromosome, the Expl value could be significantly biased and increase to even more than 100%^63^,^64^. Thus, the high contribution of each growth-related QTL in the crab is mostly because the QTLs are tightly linked in repulsion. Given that the mapping population used in QTL analysis was derived from an intercross between the northern and southern populations, this result suggests that the major growth-related QTL linked in repulsion might play important roles in the genetic control of potential heterosis in growth performance of the crab as revealed in hybrid crop^65^, which is worth to further validate.

By means of genome-wide QTL-association analysis, the markers in the linkage map were further mapped onto the genomic scaffolds in the mitten crab draft genome (unpublished data). A total of 13,198 markers were uniquely aligned to the scaffolds after removal of multiple alignments, indicating the consistency between the SNP markers, linkage map and scaffolds from the genome. As shown in Table 5, Marker253119 and Marker209500 were anchored onto Scaffold225377 and Scaffold291249, respectively. A number of growth-related genes, ligand of numb protein X 2 (LNX2), p21-activated kinase 2 (PAK2), FMRFamide receptor and octopamine receptor are located in the vicinity of the markers. LNX2 functions as an E3-ubiqutin ligase and its silencing affects the Notch and Wnt signaling pathways^66^, which play a key role in cellular proliferation and differentiation. Intriguingly, the cattle LNX2 was also identified in the genomic region associated with residual body weight gain^67^, suggesting that LNX2 may be a conserved regulator for individual growth in both invertebrate and vertebrate. PAK2, a serine/threonine kinase, acts as an effector of GTPases Rac1 and Cdc42 and is required for actin cytoskeletal remodeling, cell cycle progression, apoptosis or proliferation^68^,^69^. Full-length PAK2 stimulates cell survival and cell growth. And FMRFamide receptor is a receptor for the FMRFamide peptides, which has regulatory roles at skeletal neuromuscular junctions in insects, and is involved in muscular differentiation and growth. The inclusion of these positional candidate genes suggests a high efficiency and accuracy of this map in QTL mapping for growth traits. Further studies should be performed in order to determine the detail genetic architecture and regulatory mechanisms of these growth-related genes involved in cell proliferation and growth, and their specific association with growth traits in the mitten crab using more families and populations. The locus could be an ideal candidate target for marker-assisted selection in the crab breeding.

## Conclusions

We constructed a second generation high-density linkage map of the mitten crab *E. sinensis*. The integrated map comprising 17,680 SNP and 629 SSR markers on the 73 linkage groups represents the most saturated genetic map to date for the crab and may assist in de nove assembling genomic sequence. Three growth-related QTL were identified in a genomic region on LG53, each of which is responsible for body length, body width and body weight with high explained phenotypic variance, suggesting that they are major growth QTL. The QTL markers were further mapped onto the genomic scaffolds in the mitten crab draft genome. A number of growth-related genes were found to closely map to these loci. Consequently, the high-density linkage map can serve as an efficient platform for fine mapping of QTL and positioning sequence scaffolds, and will be valuable for better understanding of the crab genomic structure and speeding up the crab breeding program.

## Materials and Methods

### Mapping family and sampling

A F1 mapping population is derived from a cross between a female and a male parent that were collected separately from Liaohe River (northern population) in Liaoning Province and a Yangtze River (southern population) in Jiangsu Province in China^31^. The F1 progeny were reared in outdoor ponds in the mitten crab farm in Chongming island, Shanghai, China. Plastic boards were setup around each pond to prevent the crabs escaping, and the water inlet and outlet of each pond had plastic nets (mesh size: 0.17 mm) to exclude indigenous fishes and other predators from the water source and drainage channel. In the first year individuals from megalopae and juvenile (“coin-sized” crab) were raised at the high density (30–100 individual/m^2^) and in the second year from juvenile to adult at the low density (1–2 individual/m^2^)^70^. The temperature of the pond water was ambient and fluctuated depending on the season (spring and winter: 6–21 °C; summer and autumn 24–33 °C). The pond water quality was maintained with pH 7.0–9.0, dissolved oxygen >3 mg/L, ammonia <0.4 mg/L and nitrite <0.15 mg/L. Juvenile and adult individuals were randomly sampled and stored in 100% ethanol at −80 °C. Three growth-related traits, body length, body weight and body wdith, were measured as previously described^31^. Genomic DNA was extracted from leg muscles following the standard phenol-chloroform protocol^71^. Crab assays were conducted in accordance with COPE (Committee on Publication Ethics).

### SLAF library construction and sequencing

To generate a large number of high-quality SLAFs, a pre-experiment was designed to evaluate the enzymes and sizes of restriction fragments. SLAF library were constructed using the optimal pre-designed scheme with two restriction enzymes. Namely, genomic DNA from each sample was first completely digested at 37 °C with Hae III and RsaII (NEB, Ipswich, MA, USA), incubated with the Klenow Fragment (3′ → 5′exonuclease) (NEB) and dATP at 37 °C for adding a single-nucleotide A overhang to the digested fragments, and the A-tailed DNA fragment were then ligated to Duplex Tag-labeled Sequencing adapters (PAGE purified, Life Technologies) using T4 DNA ligase. PCR reaction was performed using diluted restriction ligation samples, dNTP, High-Fidelity DNA polymerase and primers: AATGATACGGCGACCACCGA and CAAGCAGAAGACGGCATACG (PAGE purified, Life Technologies). The PCR products were purified using Agencourt AMPure XP beads (Beckman Coulter, High Wycombe, UK), pooled and separated on a 2% agarose gel. Fragments of 264–464 bp (with indexes and adaptors) were excised, purified using QIAquick Gel Extraction Kit (QIAGEN) and diluted for pair-end sequencing on an Illumina Highseq™ 2500 sequencing platform (Illumina, Inc; San Diego, CA, USA) at Biomarker Technologies Corporation in Beijing. Real-time monitoring was performed for each cycle during sequencing. The ratio of high quality reads with quality scores greater than Q30 (representing a quality score of 30, indicating a 0.1% chance of an error, and thus 99.9% confidence) in the raw reads and the guanine-cytosine (GC) content were calculated for quality control.

### SLAF-seq data analysis and genotyping

The SLAF-seq data were filtered and quality assessment, and then were grouped and genotyped by the method described by Sun *et al*. Raw reads were assigned to 149 individuals according to the barcode sequences. Polymorphic SLAFs were sorted into eight segregation patterns: ab × cd, ef × eg, ab × cc, cc × ab, hk × hk, lm × ll, nn × np, aa × bb. Given that the map population is F1, the SLAF markers assorted into the pattern aa × bb were excluded in genetic map construction. Additionally, SLAF markers with average sequence depths of less than 10-fold in parents were also filtered out.

### Genetic linkage map construction

Sex-specific linkage maps was constructed for heterozygous in one parent and homozygous in the other, and marker was expected to segregate 1:1 in the F1 generation, were termed “female” or ”male”, depending on the sex of the heterozygous parent. Markers heterozygous in both parents, and expected to segregate 1:2:1 in the F1 generation, were termed “biparental” markers. The segregation type (SEG) of the female and male markers was set to lm × ll and nn × np, respectively, and biparental markers to the SEG type hk × hk. The two characters left and right of the “×” in these codes correspond to the two marker alleles of the first and second parent, respectively; each distinct marker allele is represented by a different character. The high-quality SLAF markers and 629 SSR markers in the first-generation map were selected for genetic map construction. Marker segregation ratios were calculated using the chisquare test (χ2). Markers showing segregation distortion were also integrated into the map, and the regions with more than three adjacent loci that show significant segregation distortion (P < 0.05) were defined as segregation distortion regions (SDR). Linkage grouping, marker ordering, error genotyping correction and map evaluation were performed using the HighMap method as described by Liu *et al*.^72^. Markers were divided into linkage groups (LGs), using the single-linkage clustering algorithm at logarithm of odds (LOD) threshold 4.0 and a maximum recombination fraction of 0.4.

### QTL mapping and genome-wide association analyses

QTL analysis was conducted with R/qtl software using composite internal mapping (CIM) method^73^,^74^. The significance of each QTL interval was tested by a likelihood-ratio statistic (LOD). The threshold of the LOD for significance (P = 0.05) was determined using 1,000 permutations. A QTL was determined to be significant if the LOD score was higher than the significance threshold estimated by permutation. The phenotype of morphological and growth was regarded as quantitative trait. Three growth-related traits, body length, body width and body weight, were measured and evaluated as previously described^31^. Phenotypic sex was treated as a binary trait, with female scored as “1” and male scored as “0”. Genome-wide QTL-association analysis was conducted as follow: When a QTL was captured for significant correlation with growth-related traits, the corresponding sequencing SLAF-tags were extracted and were anchored to genomic scaffolds using Blat^75^. Both homolog based analysis and de novo gene prediction was performed on the genomic scaffolds using the program Augustus^76^.

## Additional Information

**How to cite this article**: Qiu, G.-F. *et al*. A second generation SNP and SSR integrated linkage map and QTL mapping for the Chinese mitten crab *Eriocheir sinensis. Sci. Rep.* **7**, 39826; doi: 10.1038/srep39826 (2017).

**Publisher's note:** Springer Nature remains neutral with regard to jurisdictional claims in published maps and institutional affiliations.

## Supplementary Material

Supplementary Information

Supplementary Dataset 1

Supplementary Dataset 2

Supplementary Dataset 3

Supplementary Dataset 4

Supplementary Dataset 5

## Figures and Tables

**Table 1:** Statistic of SLAF sequencing data and high-quality marker depths.

Sample	Raw reads (bp)	Q30 percentage (%)	GC percentage (%)	SLAF number	Total reads number	Average depth
Female parent	6,855,402	87.13	44.39	194,887	5,771,500	29.61×
Male parent	7,192,892	86.32	44.48	200,452	6,360,679	31.73×
Average in progeny	745,247	86.39	44.43	107,758	345,020	3.15×
Total	123,599,603	85.82	44.43	235,619	/	/

**Table 2:** Statistic of the segregation patterns for SLAF markers.

Pattern	Marker number	Percentage (%)
ab × cd	3,323	15.97
ef × eg	5,714	27.47
hk × hk	303	1.46
lm × ll	6,149	29.56
nn × np	5,314	25.54
Total	20,803	100.00

**Table 3:** Summary of valid SLAF markers depths.

Sample	Marker number	Sequencing depth	Average depth
Female parent	17,680	834,252	59.34×
Male parent	17,680	697,855	49.63×
Average of progeny	11,166	47,618	4.26×

**Table 4:** Summary of 73 linkage groups for the mitten crab *E. sinensis.*

LG ID	Female map				Male map				Sex-averaged map				Female and male ratio
	SSR	SLAF	Length (cM)	Marker interval (cM)	SSR	SLAF	Length (cM)	Marker interval (cM)	SSR	SLAF	Length (cM)	Marker interval (cM)	
LG01	5	115	160.53	1.34	8	151	121.61	0.76	8	203	141.07	0.67	1.32
LG02	12	254	309.86	1.16	11	263	253.10	0.92	14	389	286.50	0.71	1.22
LG03	10	176	257.73	1.39	9	201	298.15	1.42	11	278	282.65	0.98	0.86
LG04	8	95	117.75	1.14	5	111	151.58	1.31	8	140	134.67	0.91	0.78
LG05	4	187	231.40	1.21	4	227	248.44	1.08	5	307	243.19	0.78	0.93
LG06	4	92	252.52	2.63	6	69	192.61	2.57	7	121	222.91	1.74	1.31
LG07	4	152	181.57	1.16	2	145	147.93	1.01	4	217	170.24	0.77	1.23
LG08	11	280	265.53	0.91	12	272	346.27	1.22	14	414	305.90	0.71	0.77
LG09	3	32	55.35	1.58	4	30	58.59	1.72	5	45	64.51	1.29	0.94
LG10	7	112	160.02	1.34	7	114	196.90	1.63	8	156	178.46	1.09	0.81
LG11	2	39	55.34	1.35	2	39	43.60	1.06	2	51	57.30	1.08	1.27
LG12	2	40	31.18	0.74	1	44	65.37	1.45	2	61	49.32	0.78	0.48
LG13	2	106	157.74	1.46	2	99	127.86	1.27	2	137	146.72	1.06	1.23
LG14	11	168	201.93	1.13	9	174	148.73	0.81	11	251	175.33	0.67	1.36
LG15	10	184	240.62	1.24	9	196	249.77	1.22	11	281	245.20	0.84	0.96
LG16	1	108	134.31	1.23	1	125	169.52	1.35	1	168	152.26	0.90	0.79
LG17	0	61	84.70	1.39	0	54	92.81	1.72	0	79	96.20	1.22	0.91
LG18	11	160	194.13	1.14	9	126	196.92	1.46	14	221	197.00	0.84	0.99
LG19	14	167	338.50	1.87	12	136	256.89	1.74	17	231	302.20	1.22	1.32
LG20	6	354	223.88	0.62	7	354	236.32	0.65	8	524	230.10	0.43	0.95
LG21	5	200	245.13	1.20	6	214	241.07	1.10	6	293	243.10	0.81	1.02
LG22	3	88	105.17	1.16	2	83	94.96	1.12	3	128	100.06	0.76	1.11
LG23	8	98	187.59	1.77	9	89	102.12	1.04	11	134	154.08	1.06	1.84
LG24	9	281	272.28	0.94	7	298	200.09	0.66	10	424	255.72	0.59	1.36
LG25	1	110	210.43	1.90	3	117	248.89	2.07	4	170	241.17	1.39	0.85
LG26	16	285	285.06	0.95	17	294	249.04	0.80	19	448	267.05	0.57	1.14
LG27	7	195	252.06	1.25	8	198	202.47	0.98	9	289	227.26	0.76	1.24
LG28	8	142	207.78	1.39	4	163	157.45	0.94	8	226	182.62	0.78	1.32
LG29	10	481	409.73	0.83	11	485	373.36	0.75	11	718	391.89	0.54	1.10
LG30	7	246	197.71	0.78	6	245	244.78	0.98	7	364	221.93	0.60	0.81
LG31	18	221	261.96	1.10	14	250	244.98	0.93	20	349	253.47	0.69	1.07
LG32	8	210	178.10	0.82	8	227	162.87	0.69	10	318	171.17	0.52	1.09
LG33	0	34	24.65	0.73	0	30	28.77	0.96	0	48	26.71	0.56	0.86
LG34	6	151	172.23	1.10	8	179	305.80	1.64	10	263	242.34	0.89	0.56
LG35	7	279	306.72	1.07	5	252	213.36	0.83	7	405	260.04	0.63	1.44
LG36	6	238	391.66	1.61	6	243	210.93	0.85	7	354	301.30	0.83	1.86
LG37	3	203	309.63	1.50	8	219	185.54	0.82	8	316	248.62	0.77	1.67
LG38	9	284	395.68	1.35	9	300	244.36	0.79	10	446	320.02	0.70	1.62
LG39	13	144	258.18	1.64	13	137	224.05	1.49	14	203	241.11	1.11	1.15
LG40	17	152	281.05	1.66	16	163	205.67	1.15	19	236	243.36	0.95	1.37
LG41	10	121	153.24	1.17	11	107	88.06	0.75	13	159	133.19	0.77	1.74
LG42	4	86	107.95	1.20	6	80	144.42	1.68	6	110	129.39	1.12	0.75
LG43	8	87	123.05	1.30	8	92	132.24	1.32	8	126	127.64	0.95	0.93
LG44	10	155	292.92	1.78	9	155	216.59	1.32	10	210	254.75	1.16	1.35
LG45	11	72	176.21	2.12	8	79	138.00	1.59	12	108	159.61	1.33	1.28
LG46	11	257	280.53	1.05	15	310	246.04	0.76	16	408	263.29	0.62	1.14
LG47	8	210	246.73	1.13	8	195	324.20	1.60	11	294	285.92	0.94	0.76
LG48	1	136	235.36	1.72	3	127	115.68	0.89	3	201	177.42	0.87	2.03
LG49	8	197	262.78	1.28	10	191	211.44	1.05	10	290	238.49	0.79	1.24
LG50	3	157	232.24	1.45	3	150	133.70	0.87	3	212	182.97	0.85	1.74
LG51	3	74	88.66	1.15	3	84	139.92	1.61	4	112	114.64	0.99	0.63
LG52	3	58	60.66	0.99	2	55	76.51	1.34	3	76	68.59	0.87	0.79
LG53	6	250	262.00	1.02	7	262	160.34	0.60	9	370	213.80	0.56	1.63
LG54	2	133	214.34	1.59	2	146	140.31	0.95	3	192	177.32	0.91	1.53
LG55	8	148	387.57	2.48	6	160	292.45	1.76	9	214	341.49	1.53	1.33
LG56	17	136	312.62	2.04	13	138	239.22	1.58	18	201	279.04	1.27	1.31
LG57	14	168	321.28	1.77	13	186	250.95	1.26	18	267	286.11	1.00	1.28
LG58	2	44	54.95	1.19	2	51	48.85	0.92	2	66	51.90	0.76	1.12
LG59	9	98	158.25	1.48	6	111	194.61	1.66	9	151	178.57	1.12	0.81
LG60	12	161	223.14	1.29	12	159	246.09	1.44	15	226	235.11	0.98	0.91
LG61	9	36	74.34	1.65	8	44	89.21	1.72	10	58	81.77	1.20	0.83
LG62	2	118	69.07	0.58	5	75	45.76	0.57	5	159	60.96	0.37	1.51
LG63	15	233	298.51	1.20	10	237	338.69	1.37	16	336	341.38	0.97	0.88
LG64	0	39	32.48	0.83	0	51	26.53	0.52	0	59	29.51	0.50	1.22
LG65	9	201	284.22	1.35	9	204	303.61	1.43	11	315	293.91	0.90	0.94
LG66	7	223	235.37	1.02	9	232	308.52	1.28	9	349	271.94	0.76	0.76
LG67	10	258	364.10	1.36	10	324	210.60	0.63	13	446	292.01	0.64	1.73
LG68	0	43	57.05	1.33	0	36	41.59	1.16	0	61	61.78	1.01	1.37
LG69	9	474	451.88	0.94	9	479	464.10	0.95	13	691	461.45	0.66	0.97
LG70	9	119	337.80	2.64	8	137	201.88	1.39	9	187	269.84	1.38	1.67
LG71	5	206	208.77	0.99	8	196	228.31	1.12	8	307	218.54	0.69	0.91
LG72	1	91	93.22	1.01	1	112	109.17	0.97	1	155	101.20	0.65	0.85
LG73	6	103	158.69	1.46	4	110	166.34	1.46	7	158	163.65	0.99	0.95
Total	520	11,816	15,467.37	/	508	12,191	13,817.44	/	629	17,680	14,821.92	/	/
Average	/	/	/	1.25	/	/	/	1.09	/	/	/	0.81	1.12

**Table 5:** Summary of markers significantly associated with growth-related QTL in LG53.

Trait	Marker	Position (cM)	LOD	Explained phenotype (%)	Scaffold ID	Annotation
Body length	Marker253119	169.43	59.09	95.5	Scaffold225377	LNX2; FMRFamide and octopamine receptor
	Marker01950	170.01	59.39	95.5	/	/
	Marker209500	170.60	59.77	95.5	Scaffold291249	PAK2
Body width	Marker253119	169.43	50.51	93.8	Scaffold225377	LNX2; FMRFamide and octopamine receptor
	Marker01950	170.01	50.87	93.8	/	/
	Marker209500	170.60	51.21	93.8	Scaffold291249	PAK2
Body weight	Marker253119	169.43	16.73	78.6	Scaffold225377	LNX2; FMRFamide and octopamine receptor
	Marker01950	170.01	17.07	78.5	/	/
	Marker209500	170.60	17.48	78.5	Scaffold291249	PAK2
