# Supplementary material for: A second generation SNP and SSR integrated linkage map and QTL mapping for the Chinese mitten crab Eriocheir sinensis
Source: Sci Rep. 2017 Jan 3;7:39826. doi: 10.1038/srep39826 (PMC5206627; doi:10.1038/srep39826)

Title: A second generation SSR- and SNP integrated linkage map and QTL mapping for the Chinese mitten crab *Eriocheir sinensis*

Authors: Gao-Feng Qiu [1,] *, Liang-Wei Xiong [1], Zhi-Ke Han [1], Zhi-Qiang Liu [1], Jian-Bin Feng[1], Xu-Gan Wu[1], Yin-Long Yan [2], Hong Shen [3], Long Huang [4], Li Chen [4]

1. Key Laboratory of Exploration and Utilization of Aquatic Genetic Resources Certified by Ministry of Education, College of Fisheries and Life Science, Shanghai Ocean University, 999 Hucheng Huan Road, Pudong New Area, Shanghai, 201306, China.

2. Shanghai Fisheries Research Institute, Shanghai Fisheries Technical Extension Station, Shanghai 200433, China

3. Shanghai Mudbeach Institute of Biological Resource Exploitation, Shanghai, 202150, China

4. Biomarker Technologies Corporation, Beijing 101300, China

* Correspondence author: Gao-Feng Qiu, Key Laboratory of Exploration and Utilization of Aquatic Genetic Resources Certificated by Ministry of Education, College of Fisheries and Life Science, Shanghai Ocean University, 999 Hucheng Huan Road, Shanghai, Pudong New Area, 201306, China.

TEL: 86-21-61900436; FAX: 86-21-61900436; E-mail: gfqiu@shou.edu.cn

Supplementary Information

Supplementary Figure S1-S2 and Table S1-S5, available as separate files.

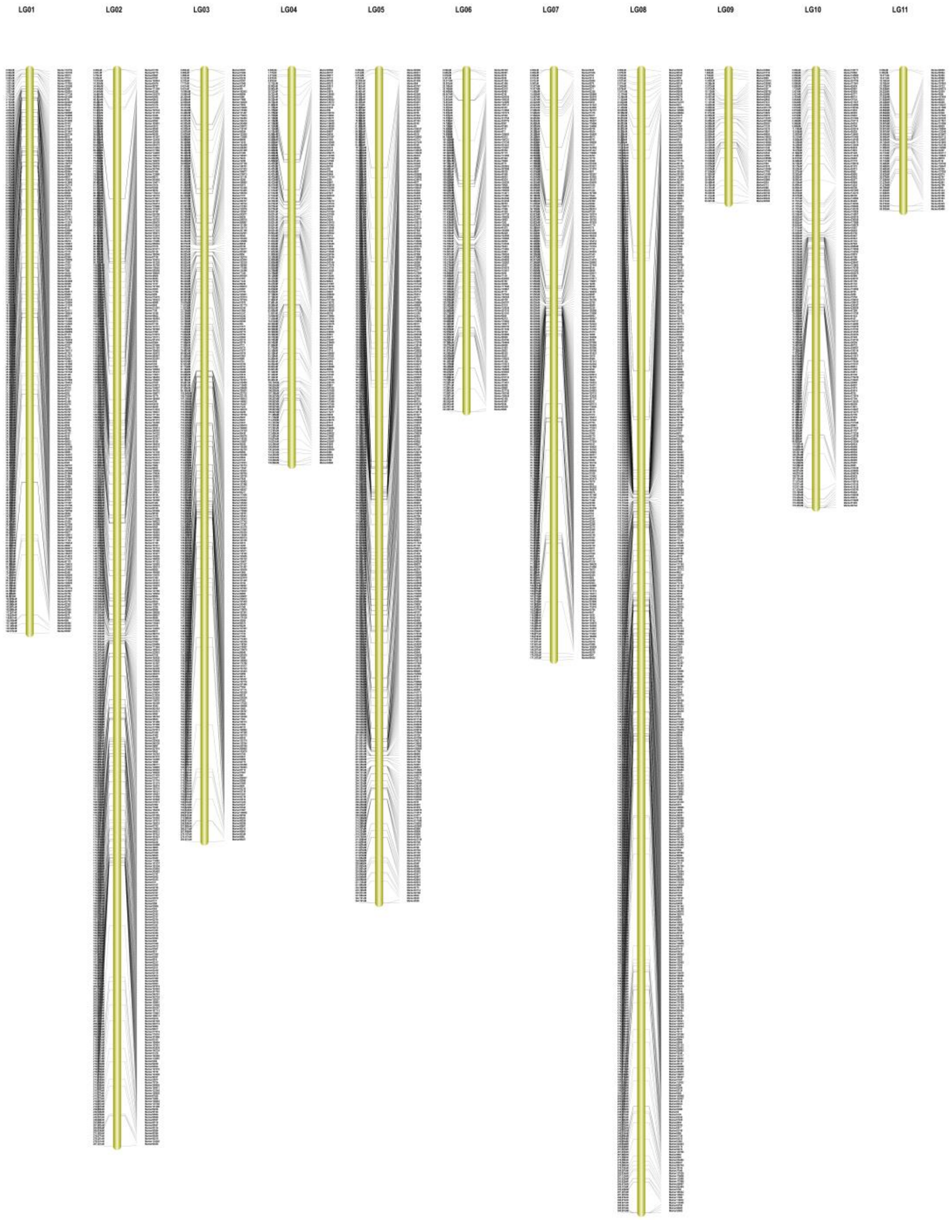

Figure S1 (A) Linkage group 1-11 of the sex-averaged genetic map for *Eriocheir sinensis*.

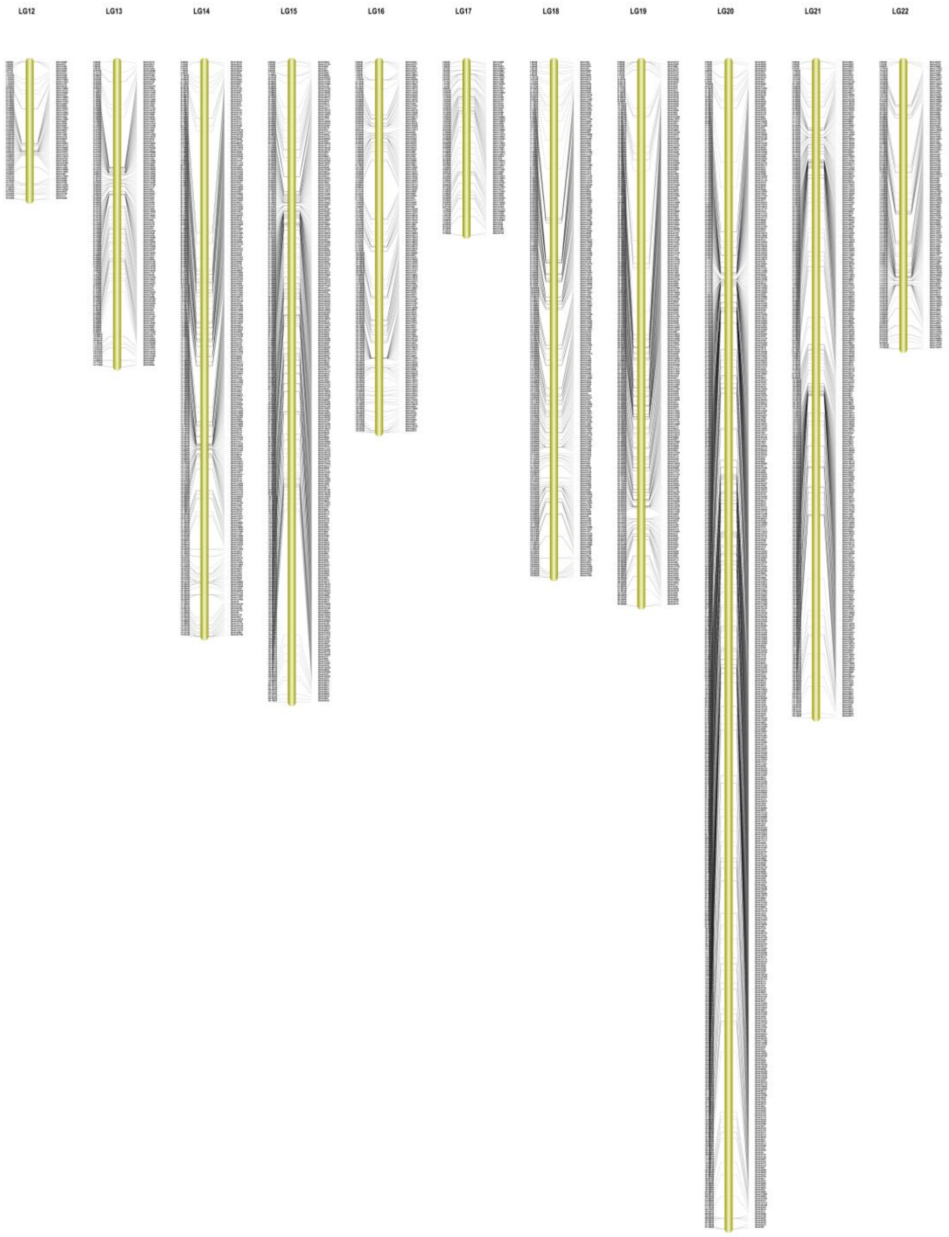

Figure S1 (B) Linkage group 12-22 of the sex-averaged genetic map for *Eriocheir sinensis*.

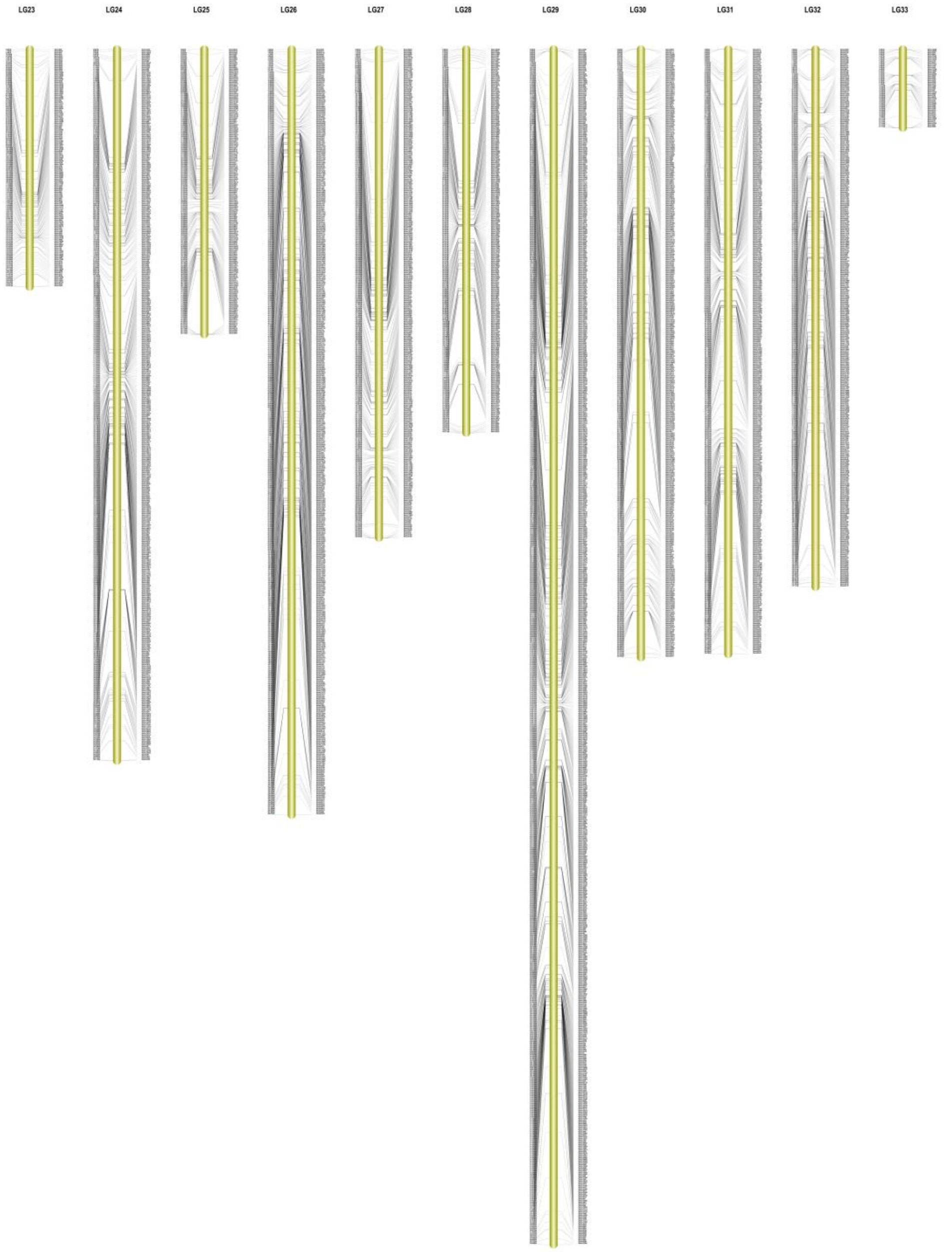

Figure S1 (C) Linkage group 23-33 of the sex-averaged genetic map for *Eriocheir sinensis*.

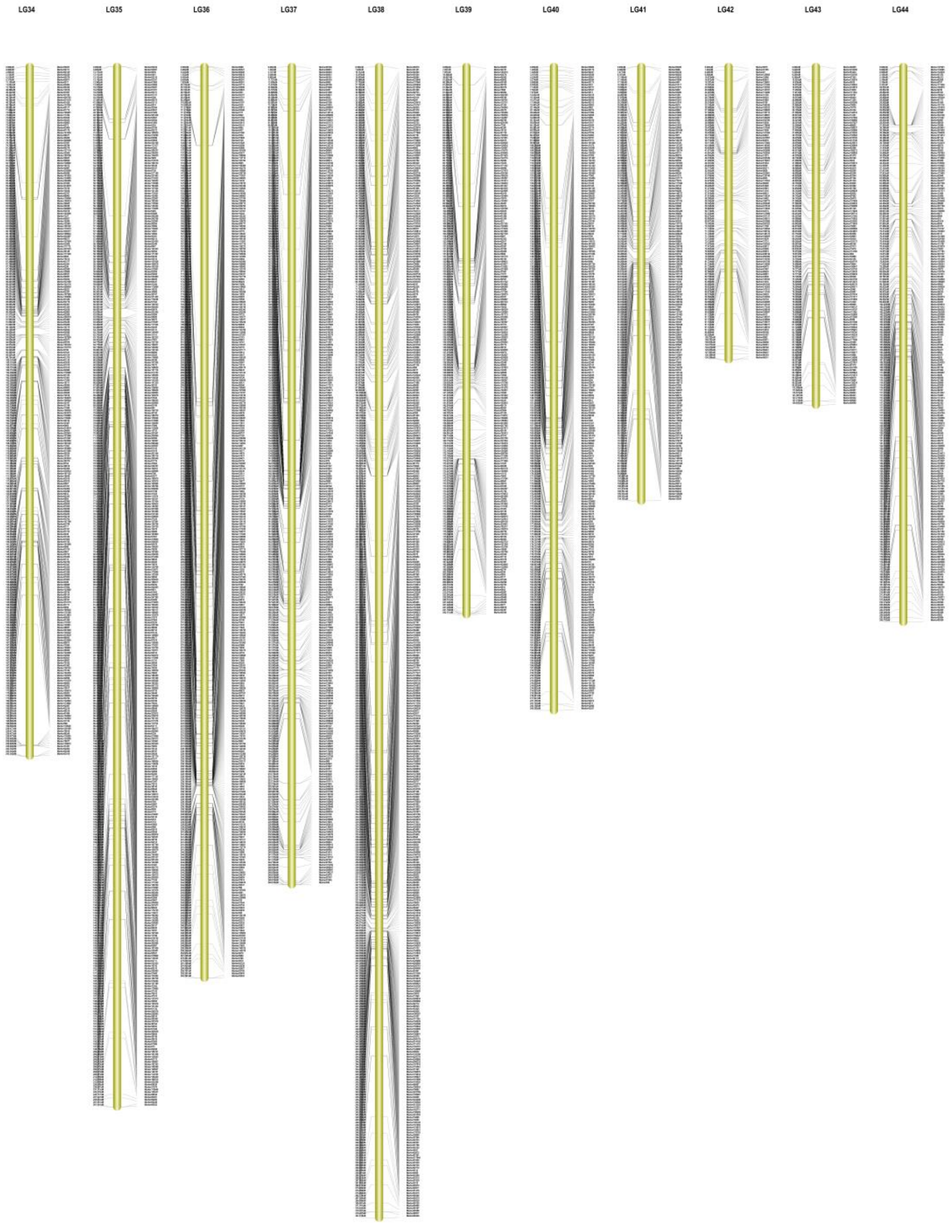

Figure S1 (D) Linkage group 34-44 of the sex-averaged genetic map for *Eriocheir sinensis*.

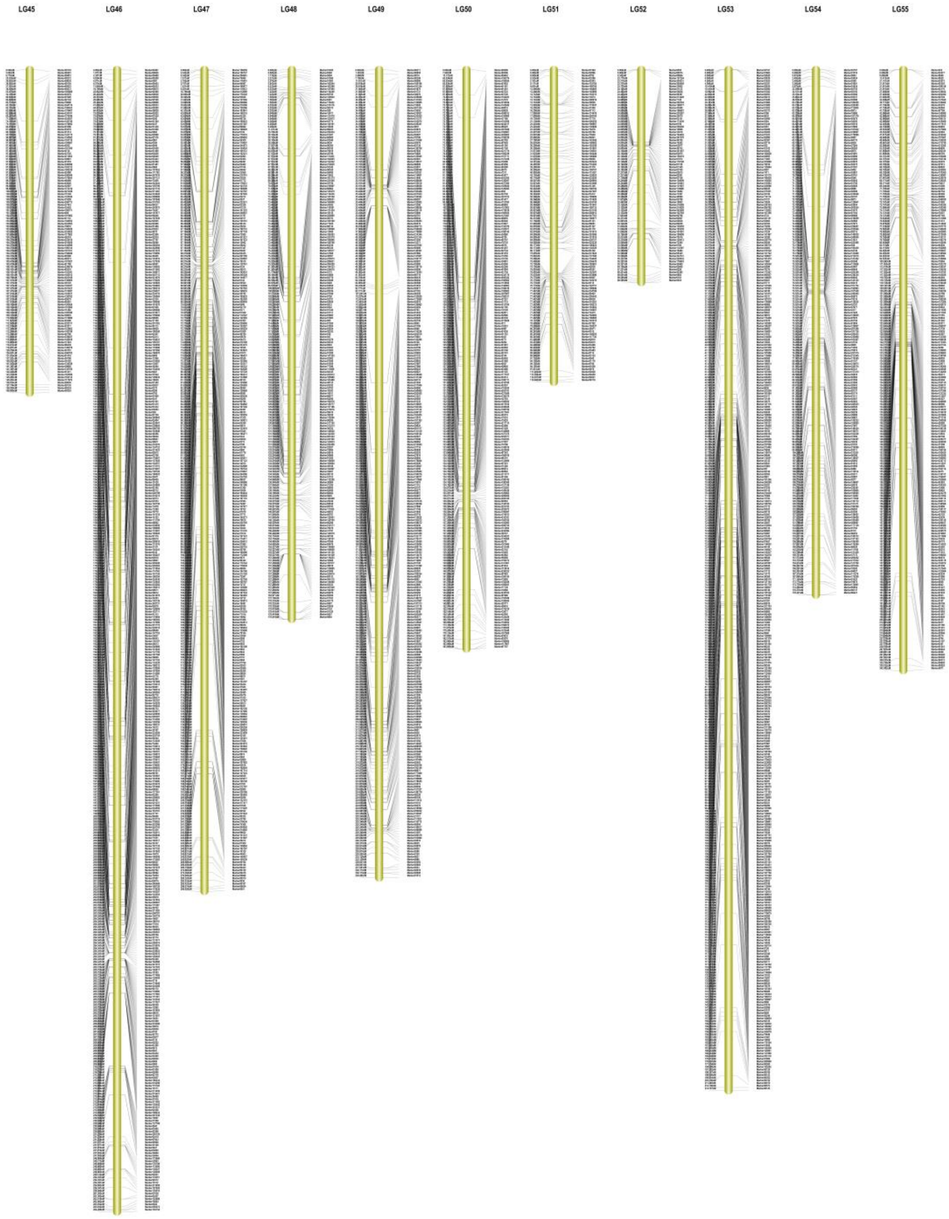

Figure S1 (E) Linkage group 45-55 of the sex-averaged genetic map for *Eriocheir sinensis*.

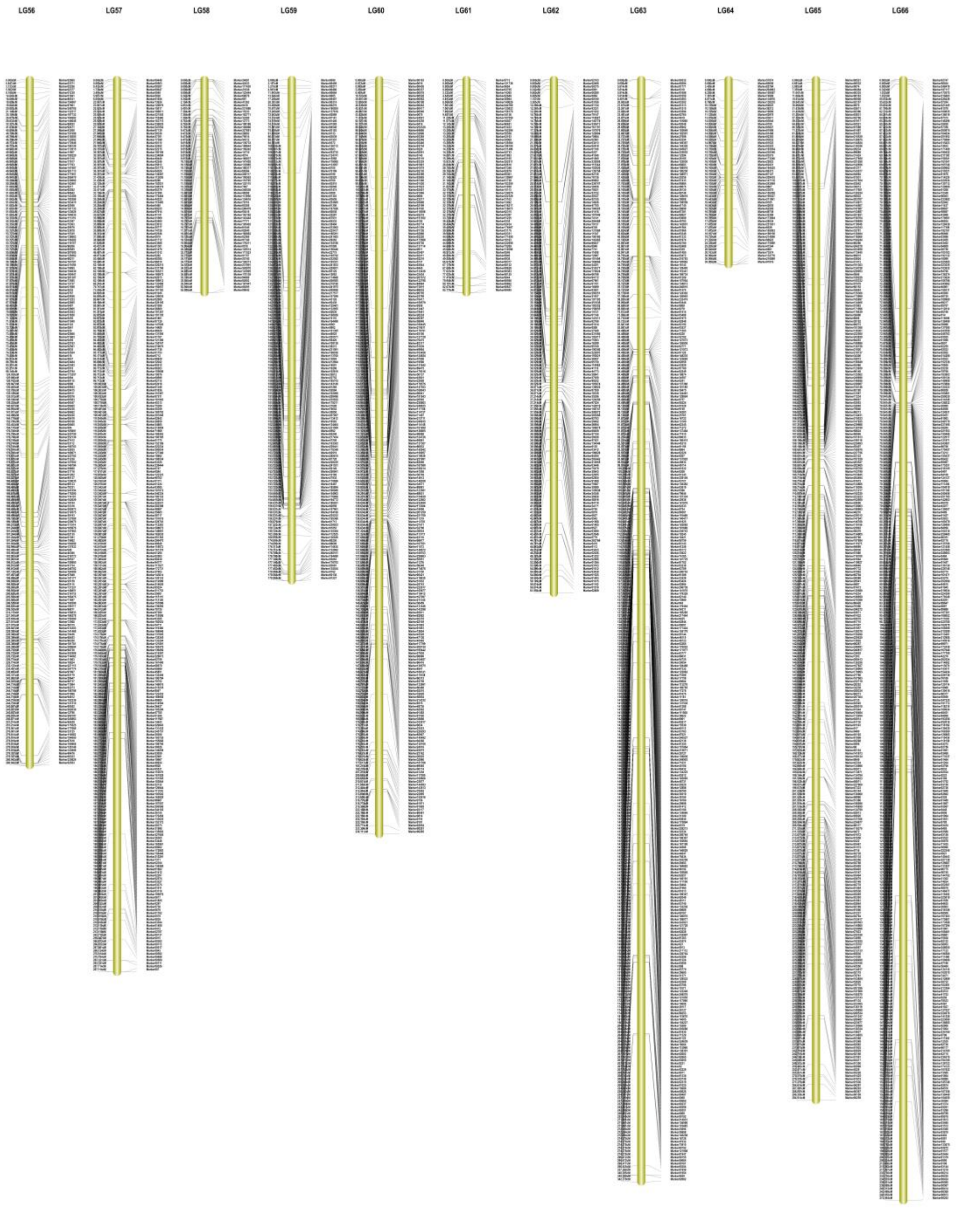

Figure S1 (F) Linkage group 56-66 of the sex-averaged genetic map for *Eriocheir sinensis*.

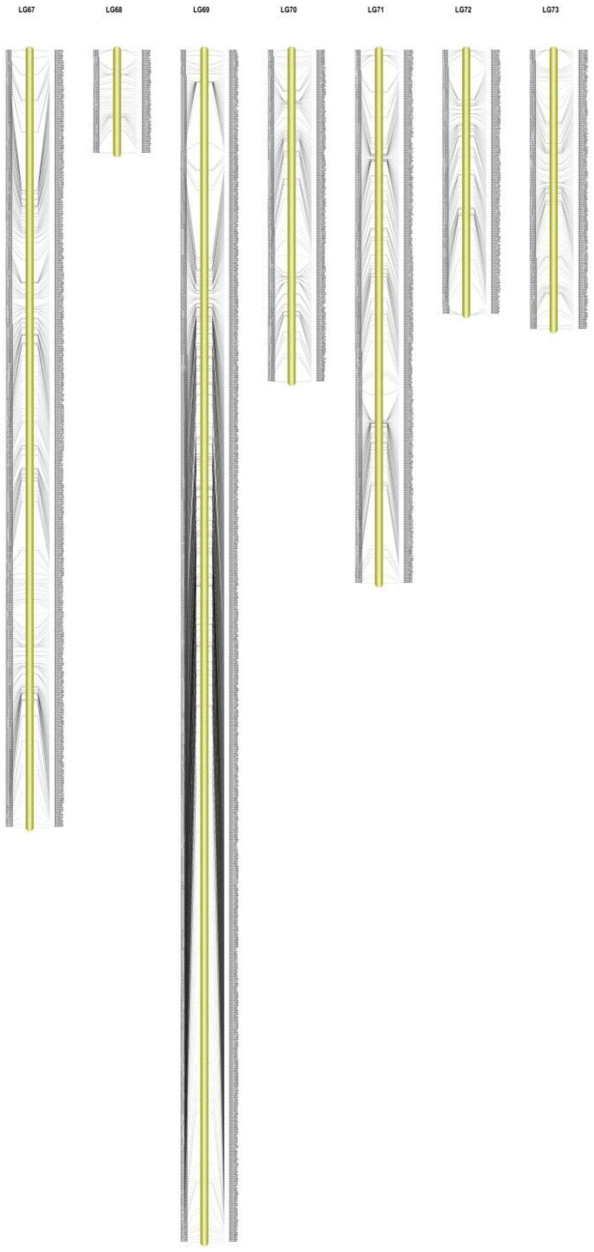

Figure S1 (G) Linkage group 67-73 of the sex-averaged genetic map for *Eriocheir sinensis*.

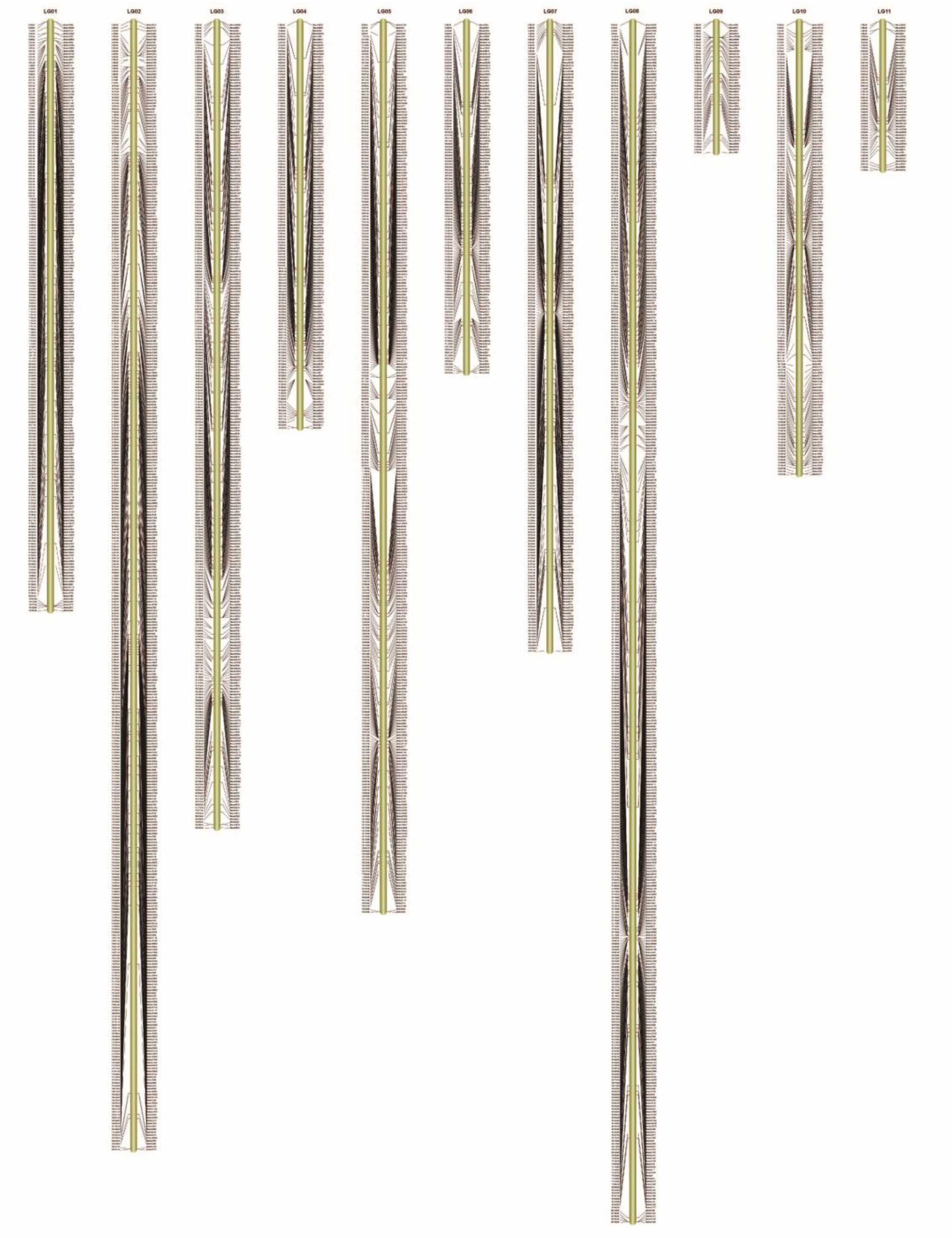

Figure S2 (A) Linkage group 1-11 of the sex-averaged genetic map created using only SLAF markers in *Eriocheir sinensis*.

Figure S2 (B) Linkage group 12-22 of the sex-averaged genetic map created using only SLAF markers in *Eriocheir sinensis*.

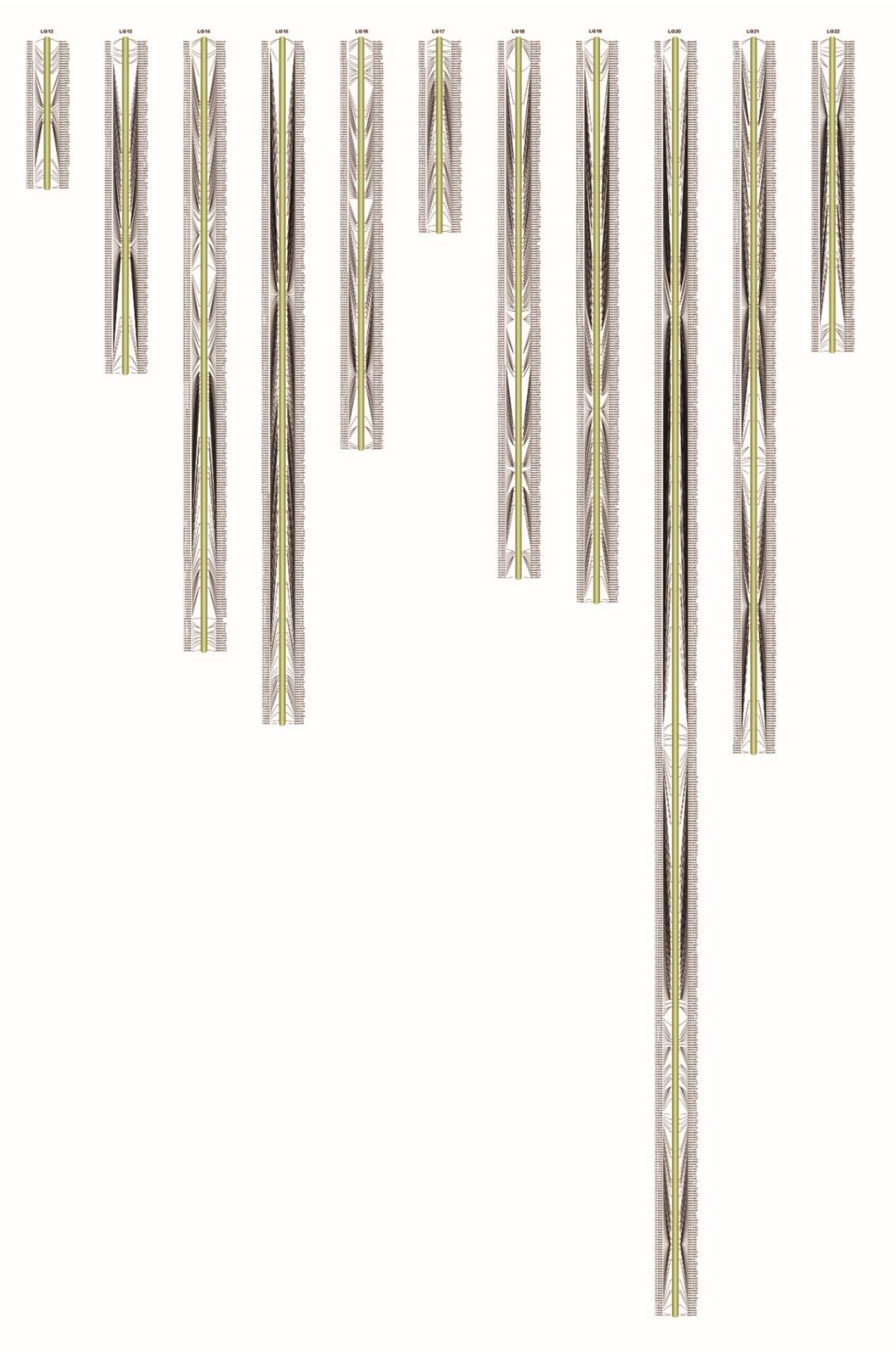

Figure S2 (C) Linkage group 23-33 of the sex-averaged genetic map created using only

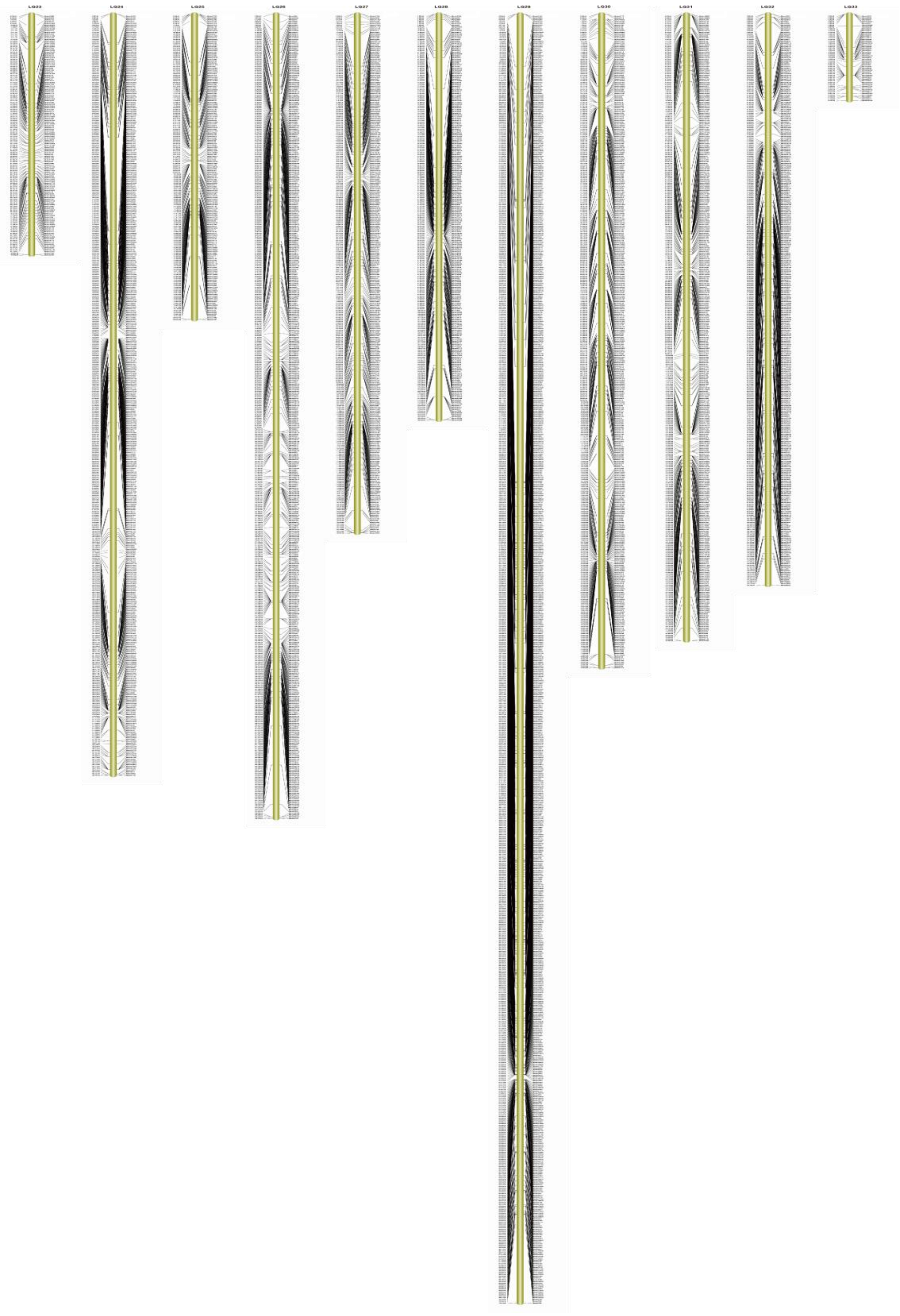

Figure S2 (C) Linkage group 23-33 of the sex-averaged genetic map created using only SLAF markers in *Eriocheir sinensis*.

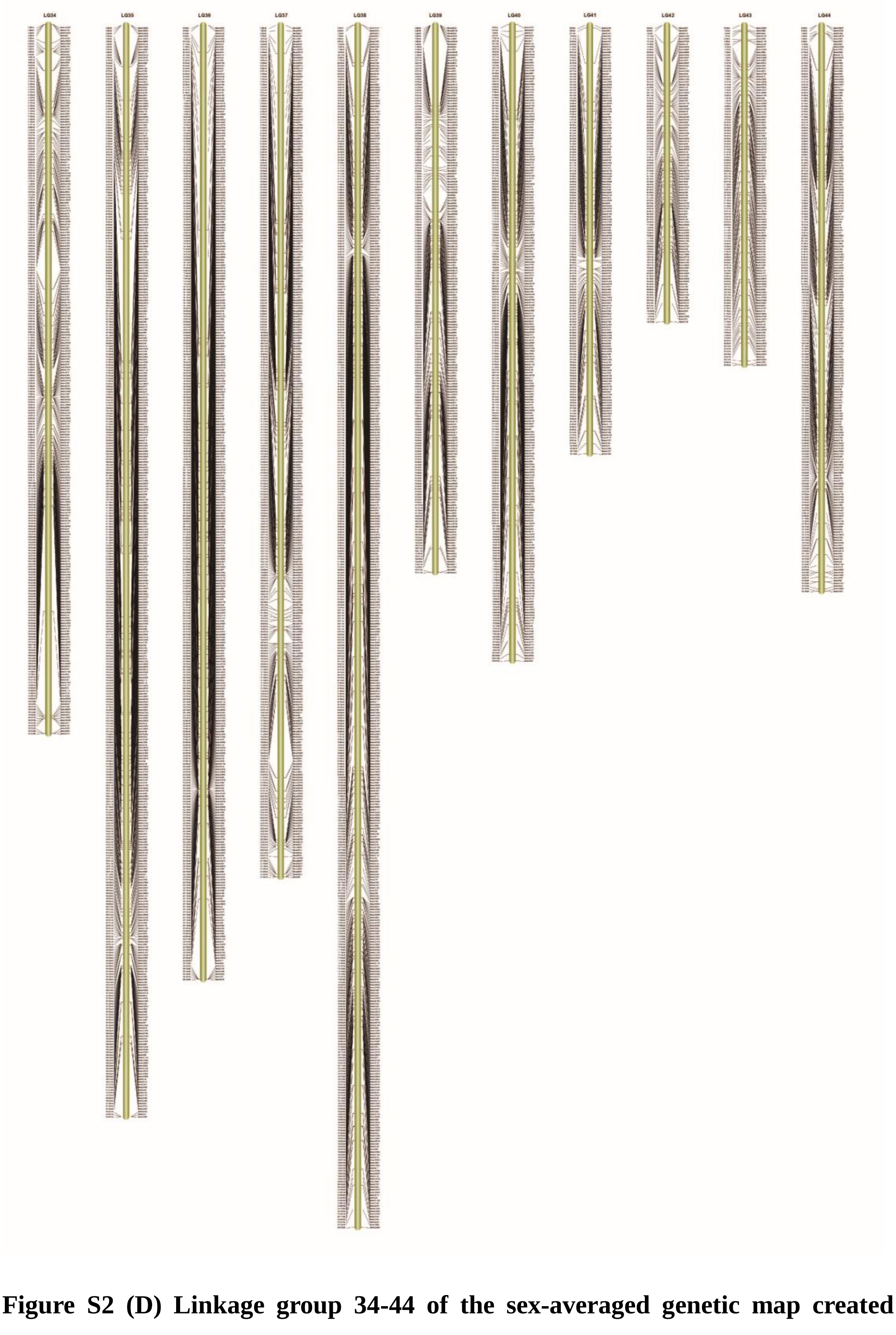

Figure S2 (D) Linkage group 34-44 of the sex-averaged genetic map created using only SLAF markers in *Eriocheir sinensis*.

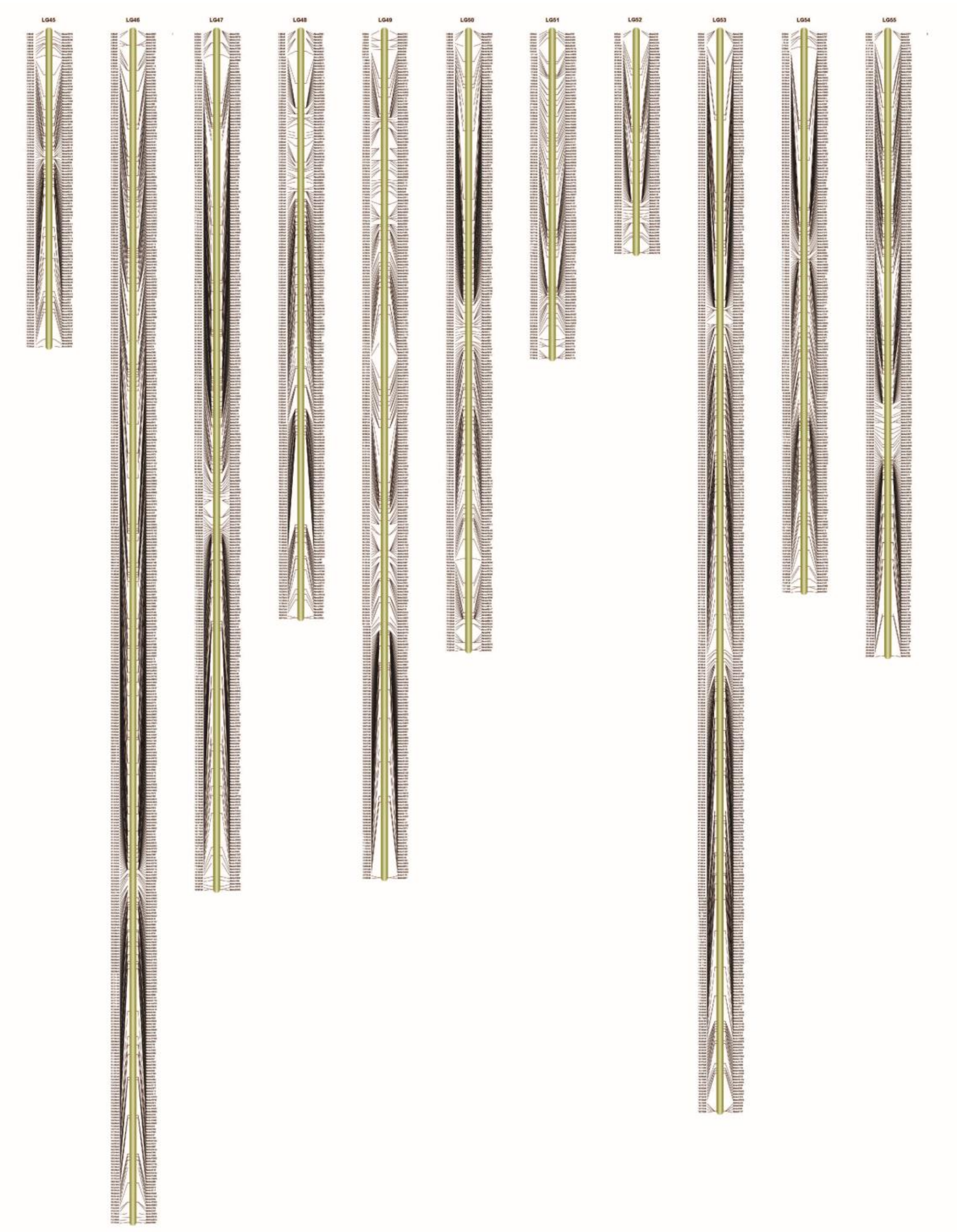

Figure S2 (E) Linkage group 45-55 of the sex-averaged genetic map created using only SLAF markers in *Eriocheir sinensis*.

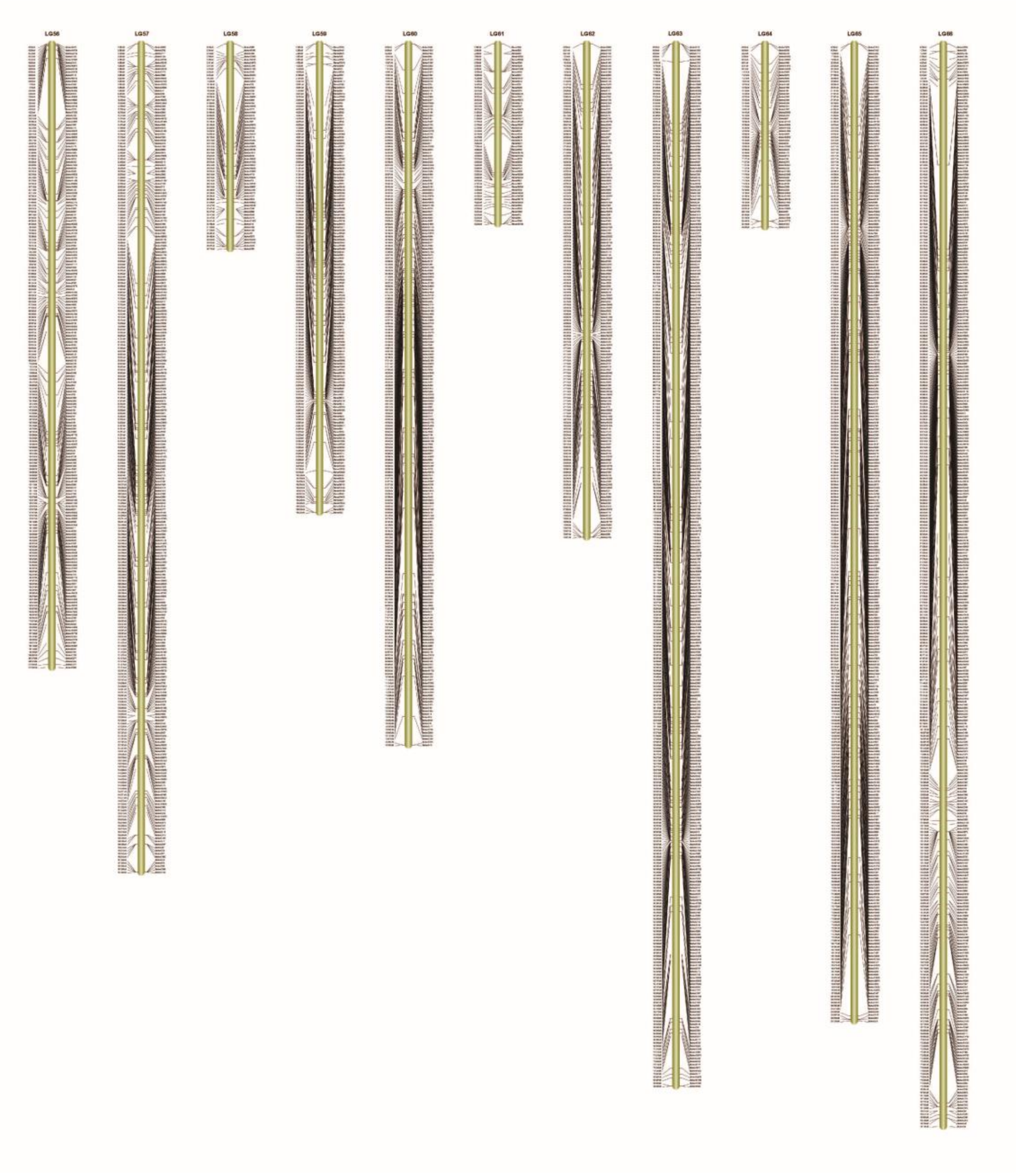

Figure S2 (F) Linkage group 56-66 of the sex-averaged genetic map created using only SLAF markers in *Eriocheir sinensis*.

Figure S2 (G) Linkage group 67-73 of the sex-averaged genetic map created using only SLAF markers in *Eriocheir sinensis*.

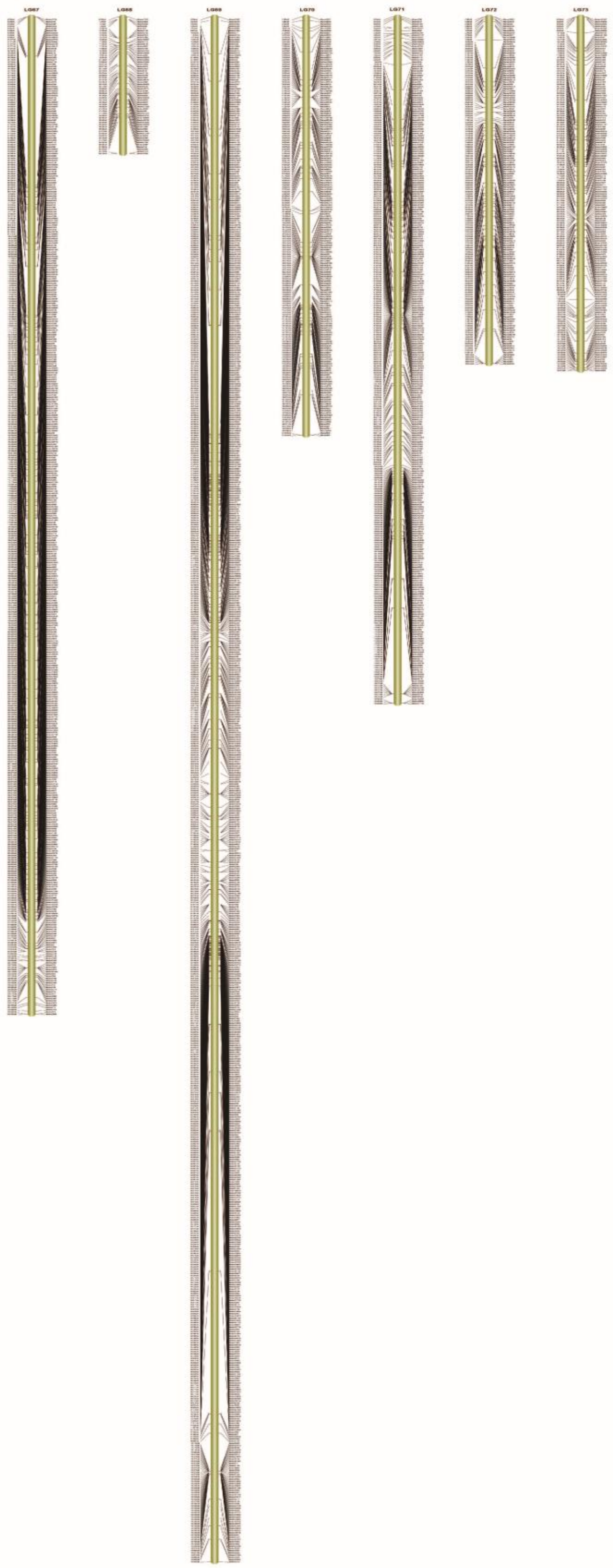

Supplement: Supplementary Information [file srep39826-s1.pdf]
